# Supplementary material for: Population screening of adults identifies novel genetic variants associated with celiac disease
Source: Sci Rep. 2025 Jun 5;15:19764. doi: 10.1038/s41598-025-04421-6 (PMC12141474; doi:10.1038/s41598-025-04421-6)
Supplement: Supplementary file 1 — Supplementary Material 1 [file 41598_2025_4421_MOESM1_ESM.docx]

**Supplementary Figure 1: QQ Plot**


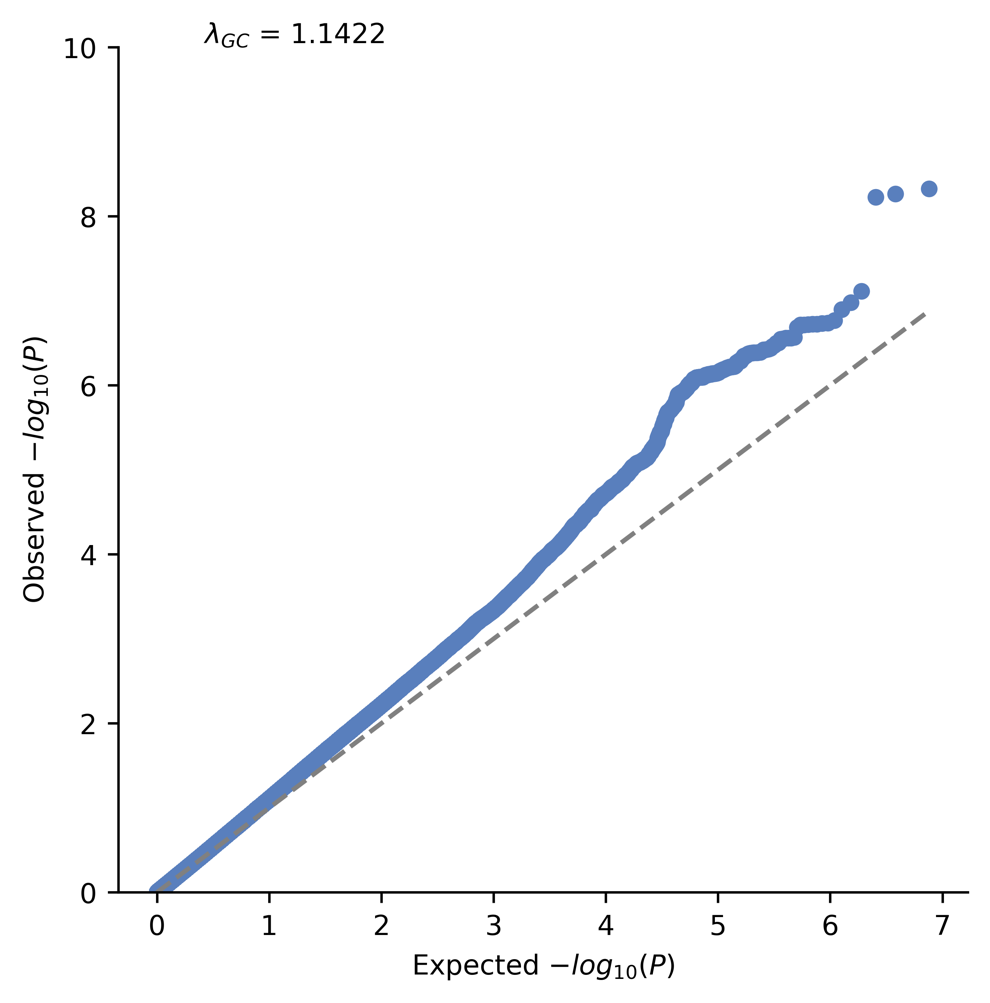

The Quantile-Quantile (QQ) plot illustrates the observed versus expected distribution of p-values from the current genome-wide association study for celiac disease in HUNT4. The x-axis represents the expected -log_10_P values under the null hypothesis of no association, while the y-axis shows the observed -log_10_P values. The diagonal dashed line indicates the line of equality, where observed p-values match the expected distribution. Deviations from this line suggest potential associations or systematic biases. Values of λ​ close to 1 suggest minimal inflation, while values significantly greater than 1 may indicate potential issues.

**Supplementary Figure 2: Subset analysis**

1.


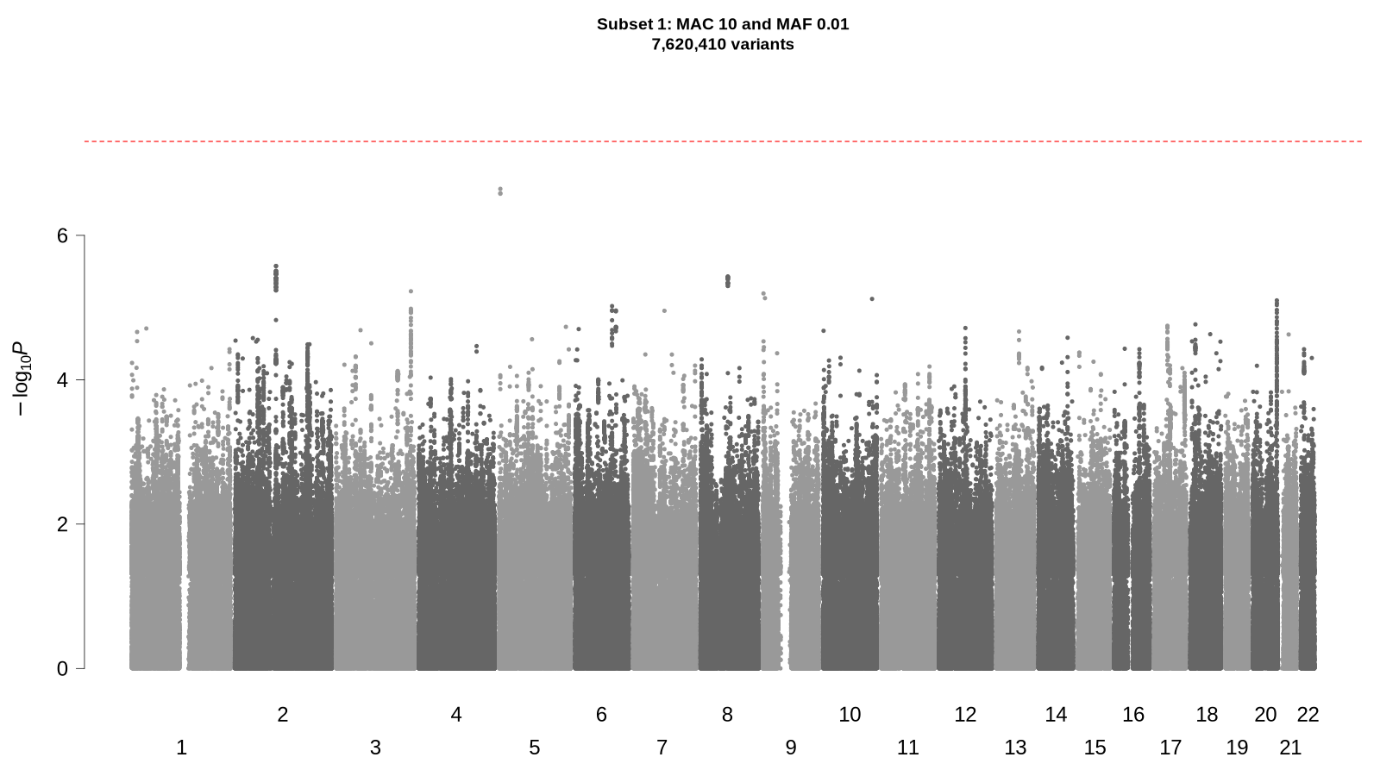


2.
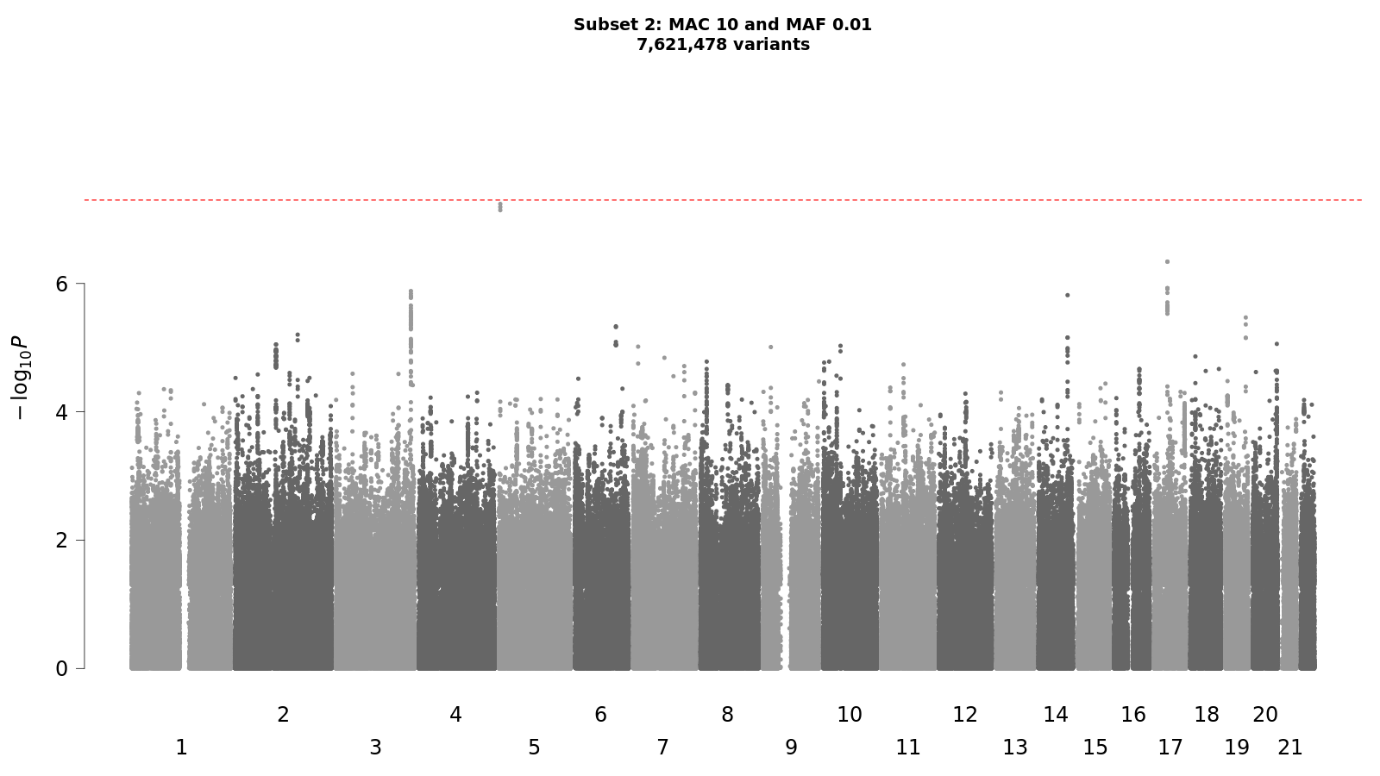


3.
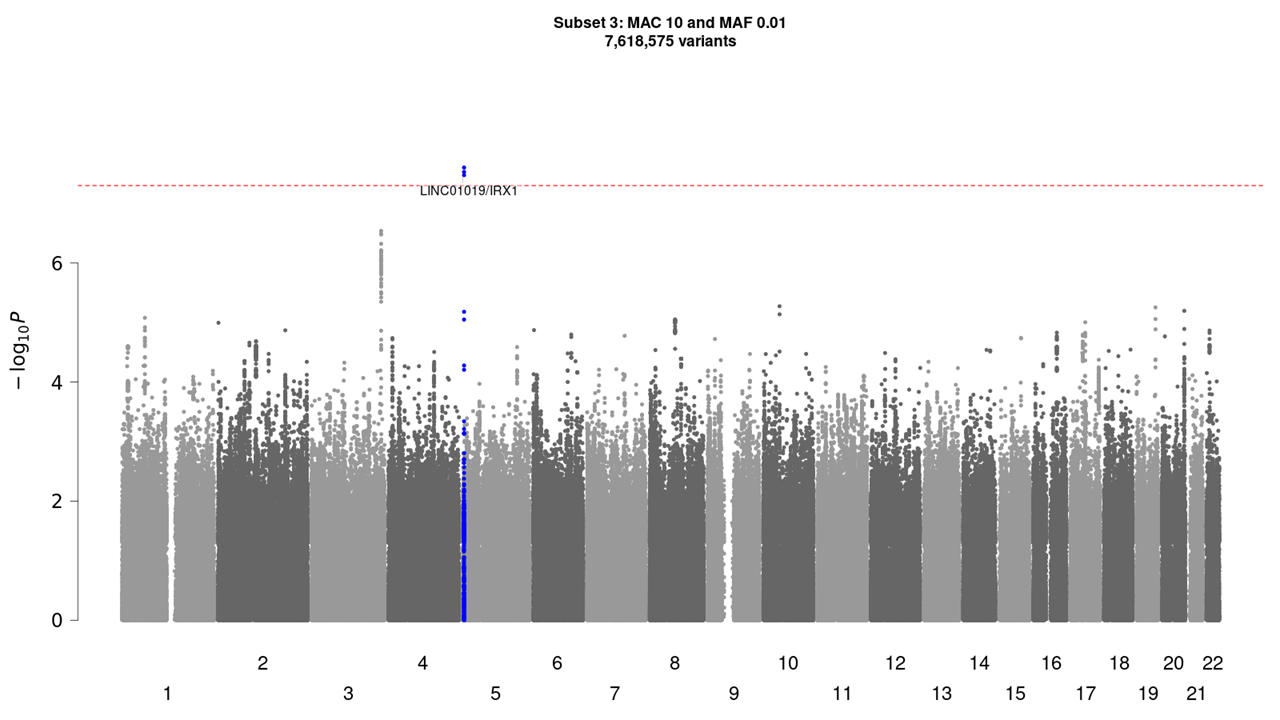


4.
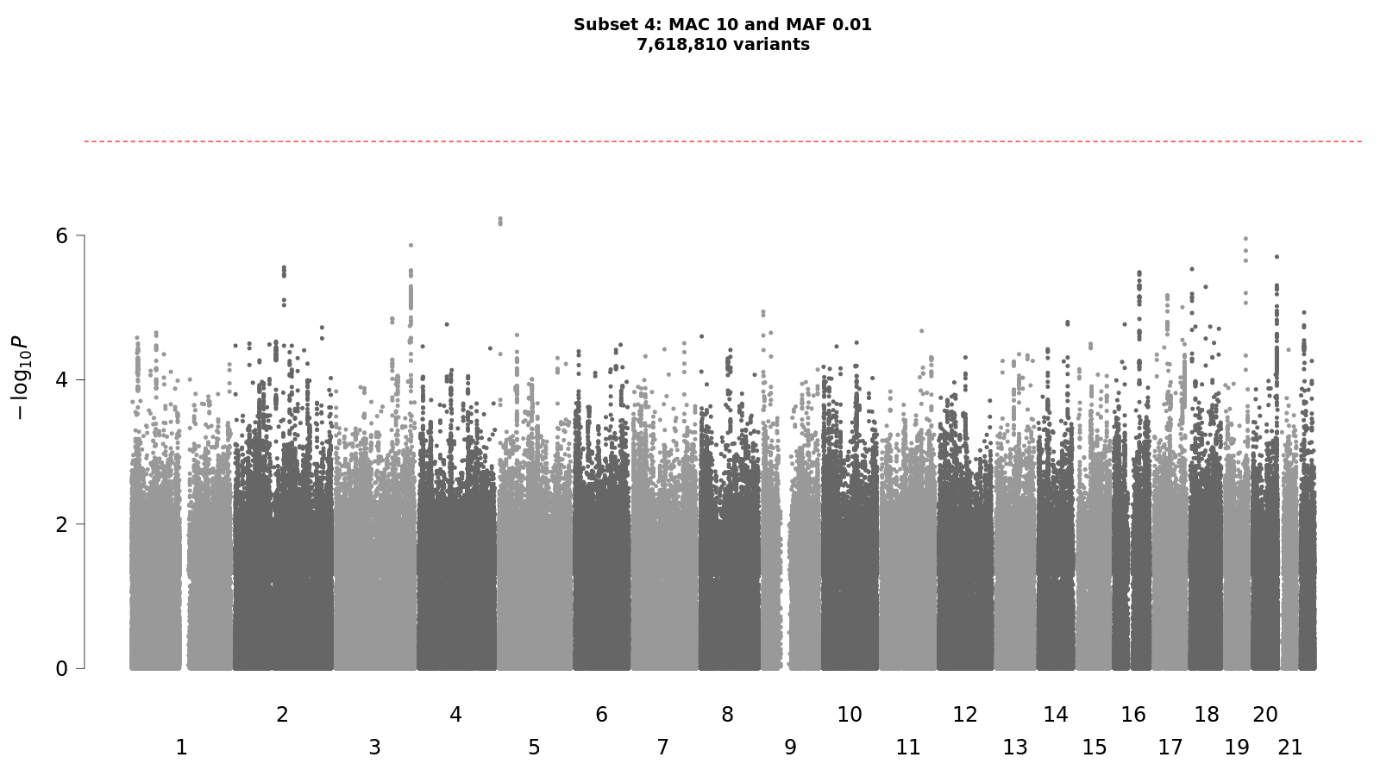


5.
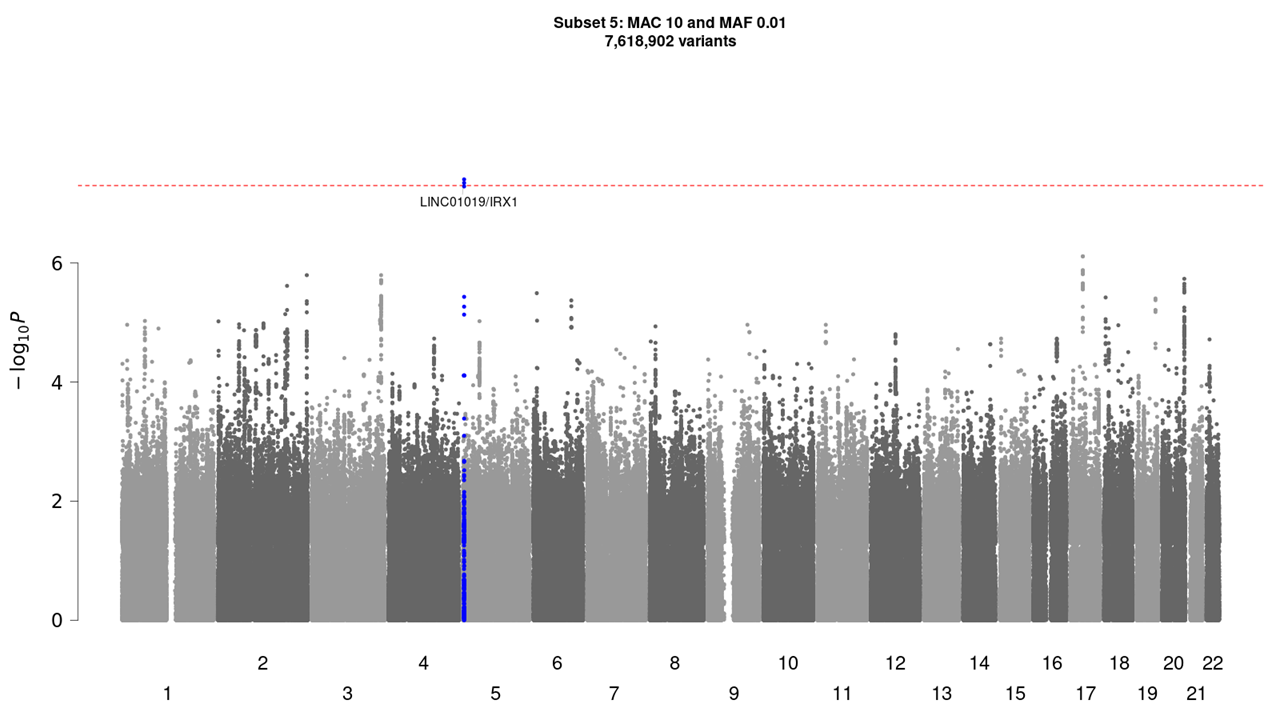


6.
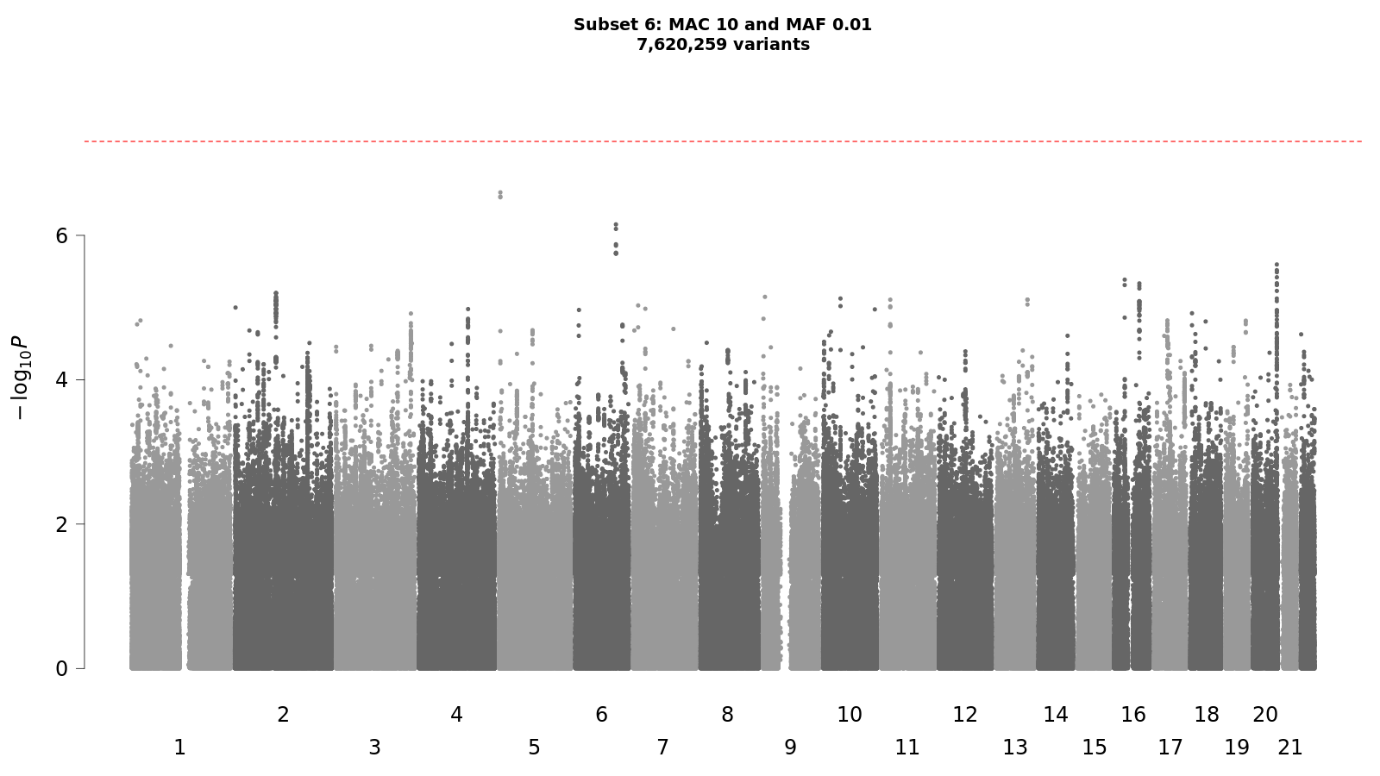


The above are six Manhattan plots for six subsets of random controls. Genome-wide significant variants are marked in blue, which grey color represents the remaining variants. The red dashed line indicate the significance threshold.
